# Supplementary material for: Biphasic Effects of Blue Light Irradiation on Different Drug-Resistant Bacterium and Exploration of Its Mechanism
Source: Biomedicines. 2025 Apr 3;13(4):868. doi: 10.3390/biomedicines13040868 (PMC12024981; doi:10.3390/biomedicines13040868)
Supplement: Supplementary file 1 [file biomedicines-13-00868-s001.zip › biomedicines-3477712-supplementary.docx]

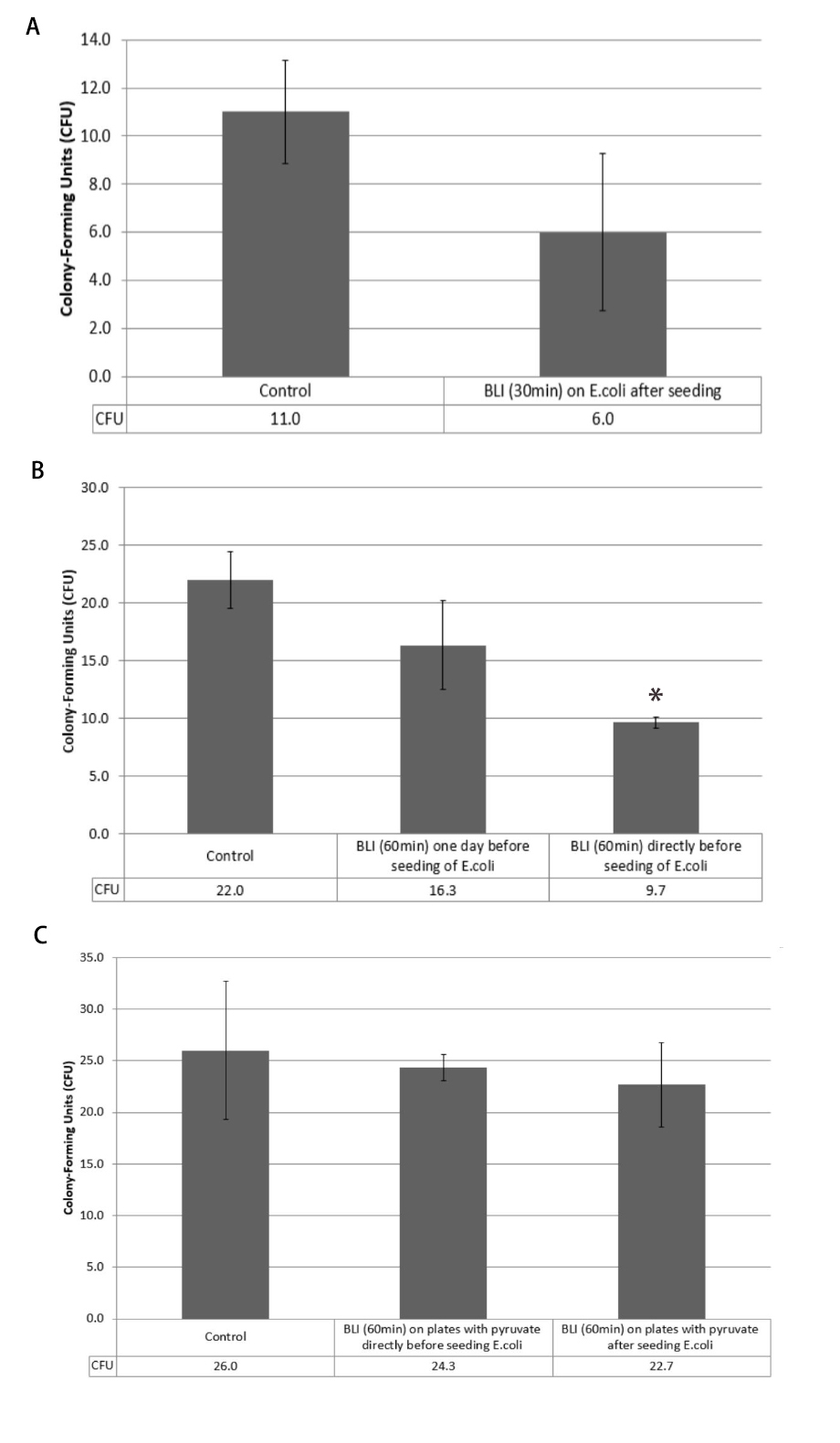


Supplementary Figure S1. (A)The effect of BLI after seeding of *E. coli* on plates compared with untreated control. Blue light treatment was performed in constant mode with a fluence of 23mW/cm^2^ and a dosage of 41.4J/cm^2^ for 30 minutes. (B) The effect of BLI on empty agar plates one day before and directly before seeding of E. coli compared with untreated control. Blue light treatment was performed in constant mode with a fluence of 23mW/cm^2^ and a dosage of 82.8J/cm^2^ for 60 minutes. (C) The effect of BLI on agar plates with pyruvate before and after seeding of *E. coli.* Blue light treatment was performed in constant mode with a fluence of 23mW/cm^2^ at a dosage of 82.8J/cm^2^ for 60 minutes. No significant difference was found among the three groups. “*” represents *p*＜0.01. Data are presented as means ± SD (N = 3 repetitions)


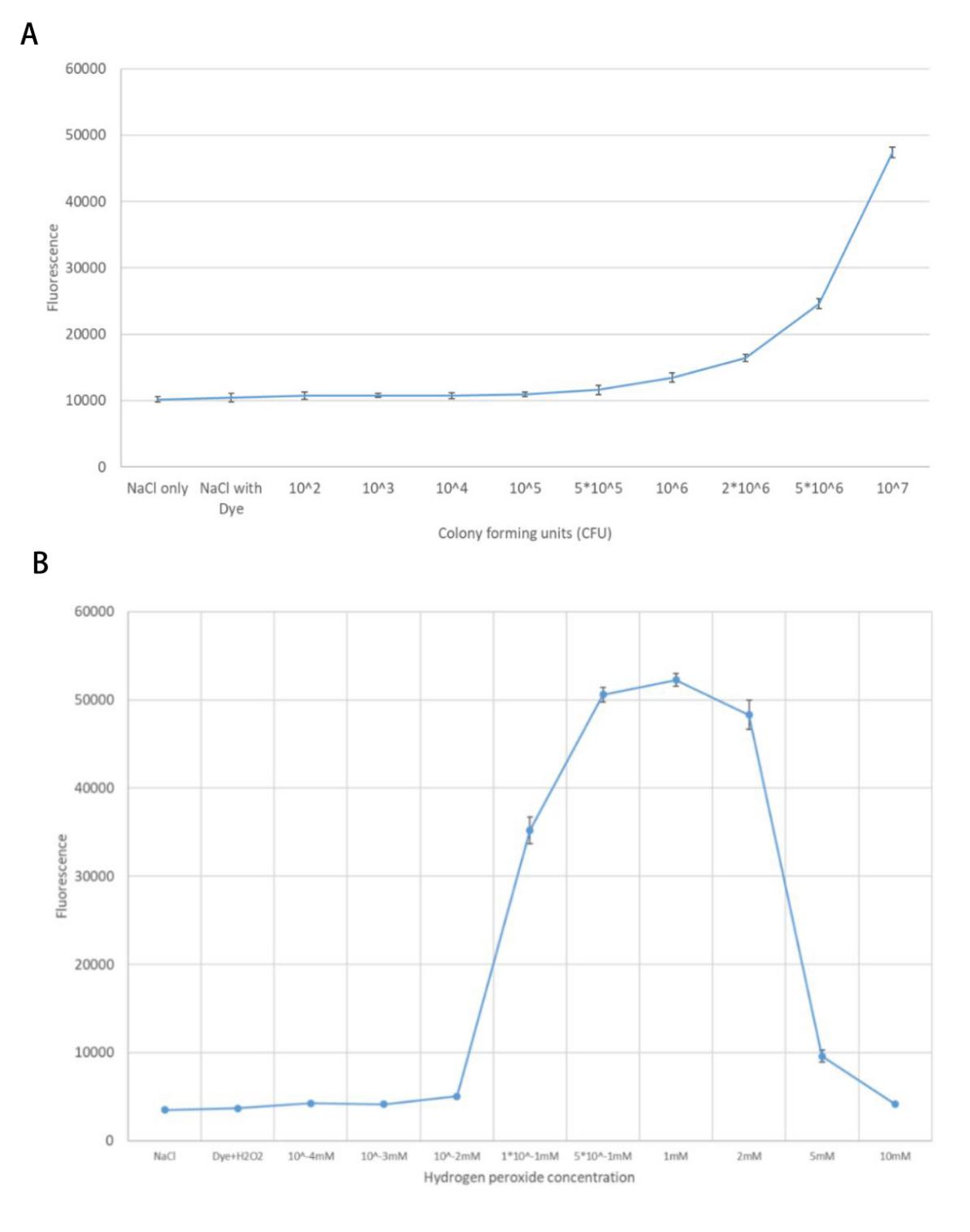


Supplementary Figure S2. (A) Changes in ROS production of *E.coli* without irradiation under different concentration of bacteria. (B) Changes in ROS production of *E.coli* without irradiation under different concentration of hydrogen peroxide as positive groups. Data are presented as means ± SD (N = 3 replicates).
